# Training community health volunteers to use mobile platform during the COVID-19 pandemic: The Kenya experience

**DOI:** 10.1017/gmh.2024.35

**Published:** 2024-03-22

**Authors:** Anne W. Mbwayo, Muthoni Mathai, Teresia Mutavi, Stella Waruinge, Noah Triplett, Tessa Concepcion, Pamela Y. Collins, Shannon Dorsey

**Affiliations:** 1 Department of Psychiatry, University of Nairobi, Nairobi, Kenya; 2 Department of Public Mental Health, University of Cape Town, Cape Town, South Africa; 3 Department of Psychology, Department of Global Health, University of Washington, Seattle, USA; 4 Department of Mental Health, Johns Hopkins University, Baltimore, USA

**Keywords:** training, task-shifting, psychosocial intervention, community health volunteers

## Abstract

This study describes an alternative to face-to-face training method for community health volunteers (CHVs) as used by a collaborative group from the University of Nairobi, University of Washington and the Nairobi Metropolitan Mental Health Team during the COVID-19 lockdown in Kenya. This qualitative study describes the experiences of 17 CHVs enrolled in a training study, required to utilize different digital platforms (Google Meet or Jitsi) as a training forum for the first time. Verbatim extracts of the participants’ daily experiences are extracted from a series of write-ups in the group WhatsApp just before the training. Daily failures and success experiences in joining a Google meet or Jitsi are recorded. Then, 17 participants, 10 women and 7 men, aged between 21 and 51 years (mean = 33), owning a smartphone, were enrolled in the study. None had used Jitsi or Google meet before. Different challenges were reported in login to either and a final decision to use Jitsi, which became the training platform. Training CHVs to deliver a psychosocial intervention using smartphones is possible. However, the trainer must establish appropriate and affordable methods when resources are constrained.

## Impact statement

Training ensures that an activity is carried out according to the requirements of a training body or group. Some training activities require face-to-face, to enable feedback and observe the bodily expressions of the trainees, which can communicate difficulties or understanding. However, sometimes face-to-face training is impossible due to various reasons, as happened during the worldwide COVID-19 pandemic lockdown. This study describes the use of smartphones to train community health volunteers (CHVs) in an informal settlement in Nairobi, Kenya to deliver a psychosocial intervention during the pandemic lockdown. The lockdown prevented the intended face-to-face training, and the team devised a creative alternative: the use of the mobile telephone to train the CHVs. Mobile phones are common in Kenya, but smartphones are needed for use of freely available applications (apps) that support training activities, for example Google Meet or Jitsi. After establishing ownership of smartphones, participants were trained on use of the Google Meet app, a new experience for the group. We describe the participants’ experiences, as captured in a WhatsApp group chat, of determining the app considered friendly to use. This process shows that with innovation and patience, training can take place solely with smartphones when face-to-face training is not possible.

## Introduction

COVID-19 presented new challenges not only to the public sector but also to the lives of individuals around the world including Kenya (Fagherazzi et al., 2020). The stay-at-home strategies for reducing viral transmission curtailed public activities around the country. Travel was canceled and alternatives to physical contact, such as digital options, were explored for education and conferences. COVID-19 also introduced challenges for training and supporting health professionals and providing mental health care. However, these challenges introduced opportunities for innovative ways to communicate, connect and carry out training and intervention delivery. We describe a method that our team used in Kenya for successful remote, mobile phone-based training of community health volunteers (CHVs). Our process offers promise and guidance for other training efforts in situations where cost or distance prevents in-person training, but high-end technology and internet bandwidth demands prevent computer-based video training. We intend to show that in the presence of limited digital literacy and the absence of computers, Android smartphones can be used when health workers need to access training with limited internet connection and only cellular service.

The work in Kenya was part of a broader multicountry pilot test of the World Health Organization (WHO) and UNICEF platform, Ensuring Quality in Psychological Support (EQUIP). EQUIP is a digital platform that provides tools to support competency‐based training in psychosocial interventions and foundational helping skills (Kohrt et al., 2020). As part of EQUIP’s activities in Kenya, our goal was to pilot test the training of CHVs to deliver a mental health intervention to distressed youth in an informal settlement in Nairobi (Mathai et al., 2023). CHVs are community-embedded lay health workers who play essential roles in health promotion and prevention in Kenya (Cometto et al., 2018), including promoting maternal and child health. Research has illustrated the effectiveness of individuals in these roles and other lay providers in delivering mental health interventions, when well-trained and supported (Mutamba et al., 2013; Gatuguta et al., 2017).

Initially, our CHVs training was to be conducted face-to-face and facilitated by experienced lay counselor supervisors and trainers from ACE Africa, a nongovernmental organization (NGO) situated in Bungoma, Kenya. However, the onset of COVID-19 and subsequent ban on travel in most countries prevented face-to-face training. Under these circumstances, we decided to switch to a virtual training mode. Given that most CHVs do not have computers or internet access in their homes, a mobile phone-based training model was pursued, where the participants could access the internet through prepaid data bundles (“airtime bundles”) provided by our team.

Globally, there were an estimated 5.1 billion unique mobile phone users in 2018. Mobile phone use in low-to-middle-income countries has increased with about 82% of persons from 33 of 54 countries in Africa owning a mobile phone and those in urban settings having a three times odds then their rural counterparts (Okano et al., 2022). WHO reports that mobile phones have reached more people than any other mode of communication technology (WHO Global Observatory for eHealth, 2011). Kenya is reported to have a mobile penetration rate of 87%, with the penetration being higher in urban areas, such as Nairobi, where the training was conducted and most training participants live (Ndung’u et al., 2019).

CHVs and other healthcare providers increasingly use mobile phones for health interventions (Winters et al., 2018; Marongwe et al., 2022; Mureithi et al., 2023), and a number of researchers have called for greater exploration of how mobile phones may be used to increase access to mental health care specifically or support the delivery of mental health care (Naslund et al., 2017, 2019; Hoeft et al., 2018). A growing body of research has examined how mobile technology can support lay mental health providers, including through supporting training (Shields-Zeeman et al., 2017; Naslund et al., 2019), aiding in clinical diagnosis and decision-making (Maulik et al., 2017; Diez-Canseco et al., 2018) and facilitating supervision (Gureje et al., 2015; Rahman et al., 2019; Xu et al., 2019; Triplett et al., 2023). However, to our knowledge, no studies have attempted to train lay providers in mental health interventions entirely through a mobile phone application.

There is tremendous potential in employing mobile phones to train lay providers, which could drastically increase the reach of training programs by reducing barriers of travel and hardware requirements of other technology-based training models (e.g., computers and Wi-Fi). However, Bakibinga et al. (2020) point out some obstacles in the implementation of digital health solutions with CHVs in Kenya: not typically having a smartphone and slow attitudinal changes regarding digital health solutions, especially among older CHVs. The authors also note that previous exposure to digital technology made mobile phone use easier (Bakibinga et al., 2020). Other studies have also underscored the need for training CHVs on how to use smart phones prior to beginning phone-based work (Shields-Zeeman et al., 2017).

The mental health community needs guidance on how to address and overcome barriers associated with digital health solutions to fully realize the potential of these technologies to support mental health care delivery and contribute to closing the global treatment and care gap. The present paper documents the process of using mobile phones (i.e., smart phones) to train CHVs from an informal settlement in Nairobi to deliver a mental health intervention. We include illustrative quotes and interactions among CHVs and trainers, extracted from WhatsApp communications, that provide insight into CHVs participant experiences with each step of our process. We hope that this process can provide a guide for others to successfully deliver training in contexts limited to mobile phone technology.

## Procedures

### Ethics

Ethical clearance for all study activities was obtained from the WHO Ethical Review Committee, the Kenyatta National Hospital-University of Nairobi Ethical Review Committee (KNH/UoN ERC), and the University of Washington Institutional Review Board. Our team sought and obtained ethics approval for our transition to virtual/mobile phone training and virtual intervention delivery as soon as physical, in-person meetings were discontinued in Nairobi. CHVs received the consent form sent through WhatsApp enabling review prior to a thorough discussion with the first author, who called each CHV providing time for questions and discussion prior to CHVs providing consent.

### Study setting and study partnerships

The EQUIP Kenya team focused on one community center that serves Mukuru, one of the larger, sprawling informal settlements in East Nairobi. Like other informal settlements, Mukuru is characterized by high levels of poverty, unemployment and poor infrastructure. We partnered with Mukuru Promotion Centre; a local NGO run by the Catholic Church. The Centre offers educational, health, vocational and social services to the communities, and we worked with CHVs that were engaged through the center.

The CHVs work in the health sector as part of the community health workforce, being supervised by the Community Health Assistants. They offer health outreaches in the community, based on health challenges such as; nutritional assessments and supplements, health education on water, sanitation and hygiene, family planning, HIV and TB services such as follow-up of clients, perinatal health services such as ensuring mothers attend antenatal and postnatal clinics, folic supplement and immunization. They are not specialized in mental health. Currently, all the CHVs are given a monthly stipend by the government but they mostly rely on working with the different implementation and research partners in various areas where they are contracted to work to support the services. They have limited or no experience delivering mental health interventions. EQUIP Kenya included a partnership between the University of Nairobi, Nairobi City County (NCC), Ace Africa Bungoma, citiesRISE and the University of Washington (Mathai et al., 2023).

### Participants and recruitment

Participants were 17 CHVs. NCC Mental Health Team lead (SW) guided the selection criteria for CHVs. Through a series of meetings, we decided participating CHVs should be registered as CHVs in NCC and already working in the identified study sites. In addition, each had to be in possession of a smartphone. Through SW, the CHVs were approached and told about the study. Those who were interested and wanted to know more about the study gave their telephone numbers to be called later. The study team telephoned 19 CHVs and recruited 18. One CHV, however, dropped out due to non-study-related reasons. The remaining 17 continued until the end of the study, participating in all training activities and delivering the mental health intervention. Participants comprised 10 women and 7 men ranging in age from 21 to 51 years (mean = 33). The majority (14) had achieved 12 years of education (range 10–12).

### Training

**Figure 1.**
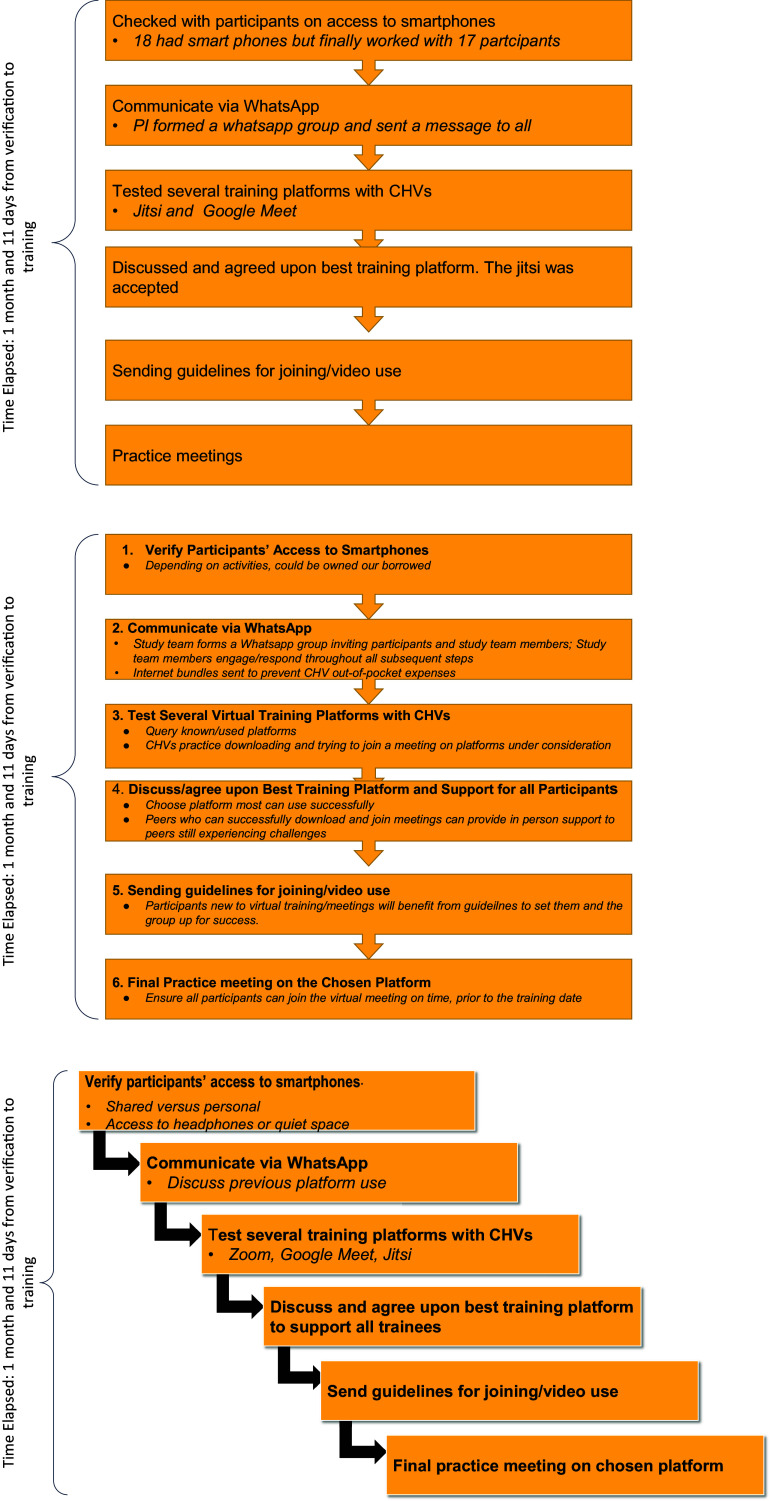
Pretrainging steps.

Our process involved six low-ask/burden pretraining steps with CHVs, over 6 weeks, to support a successful mobile phone training and a follow-on supervision plan (Figure 1). In each section, we provide details on the goal of each activity and how our team carried out the activity. Each pretraining step involved questions of CHVs that might take merely minutes (e.g., Step 1) and others involved a task that might take 1–2 h (Step 3, Step 4 and Step 6). We provide illustrative quotes from CHV training participants, based on extraction from the group WhatsApp communication, that provide their perspectives on acceptability and feasibility. In group WhatsApp communications, any needed translation from Kiswahili to English was done by the Kenyan research team, who were fluent in Kiswahili and English. The Kiswahili used was the ordinary spoken Kiswahili in Nairobi and generally in Kenya but not the taught/academic Kiswahili. We use numerical codes for CHVs and include no identifying information (e.g., no names, gender, location or other potentially identifying information).

**Step 1. Verify participants’ access to smartphones**. While mental health professionals and trainers may have regular access to computers, many lay counselors may not. With a smartphone, however, they can see written material, view the trainers, practice learned skills with other trainees, and have their practice observed by trainers. Before beginning, it is essential to confirm that trainees’ own smartphones or have access to a smartphone they could borrow. Prior to moving to Step 2, our EQUIP team confirmed smartphone access for all participants.

**Step 2. Communicate via WhatsApp.** To carry out the multistep pretraining process designed to ensure training success, teams will need a method for communication between trainers’/team members and training participants. Given the widespread use of WhatsApp and likely trainee fluency with this platform, WhatsApp is a viable communication platform to consider.

In EQUIP Kenya, we chose WhatsApp. The NCC Metropolitan Mental Health Unit contact asked participating CHVs via SMS if they used WhatsApp, and, as anticipated, all participants were WhatsApp users. The study co-PI opened the WhatsApp group on September 23, 2020 (MM). Importantly, internet bundles were then sent to CHVs by the co-PI to cover the costs of study- and training-related communication, so that CHVs did not personally bear the cost of communication and so that they would fully engage, knowing the study bore the data support costs. With an established WhatsApp group for the study, we were all to relay all pertinent communications and training activities to all CHVs at once, streamlining communications and reducing the likelihood that some CHVs would not receive information. The WhatsApp group also facilitated CHV trainee communication with each other.

**Step 3. Test several virtual training platforms with CHVs.** To successfully conduct provider training using mobile phones, and particularly when trainees may be less familiar with virtual platforms, it is important to find a platform that the trainees can use successfully and that does not require high bandwidth internet that would be costly and quickly consume provided data bundles. Letting trainees try out platforms with plenty of practice time prior to the actual training allows identification of the best platform for participants. It also allows participants to try out the different functions of different platforms pretraining (e.g., mute/unmute, chat, video/audio). It is essential to allow ample time for downloading platforms and attempting to join, across multiple days. It also is essential that study team members monitor and respond to questions, concerns and challenges in a time-sensitive way, as well as create an environment that encourages the participants to support each other.

In our project, we wanted to start by determining if participating CHVs had any experience in a virtual platform that they could successfully access on mobile phones to conduct the training. We posted the question on the group WhatsApp. Two CHVs reported having used Zoom and two had used M-learning and chat (for sending messages); no other platforms were familiar to participants. The group chat responses suggested that all CHVs were eager to learn. We could not use zoom as we did not have a subscription and the study did not have a budget for it.

**Figure 2.**
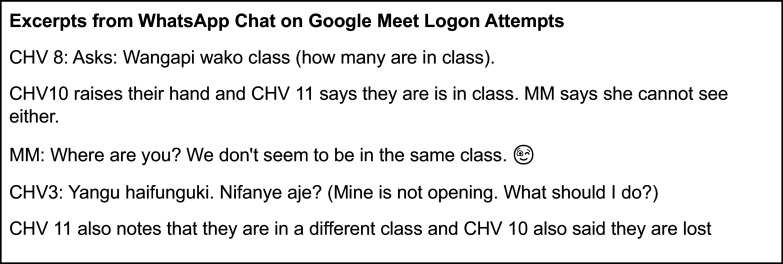
Excerpts from WhatsApp Chat on Google Meet Logon Attempts.

The CHVs were asked to download the Google Meet. On October 6, 2020 (1 month before training), a Google Meet link was sent to the group chat, proposing that participants join the Google meet link at 2 pm that same day. This “practice meeting” made it clear that Google Meet was challenging. At the meeting time, the co-PI (MM) and one co-investigator (AM) logged in but no one else had logged in. On WhatsApp, five CHVs said they were logged in but MM and AM could not see them. In the end, only four participants managed to log on to the scheduled Google Meet. The group WhatsApp chat facilitated understanding of the challenges encountered in real time, by the CHVs trying to join on Google Meet (see Figure 2).

The co-PI used WhatsApp to ask everyone to end their attempts to get into that Google meet meeting, and sent a new Google Meet link. However, even with a new second link and a third link, we were still unable to have all CHVs join. At least one CHV still said that their Google Meet link could not even be opened. Having the WhatsApp chat, however, allowed CHVs to ask questions of the study team and each other, but it was not sufficient to support all 17 getting onto Google meet. Given the ongoing challenges, CHVs were asked to abandon Google Meet and instead try to download Jitsi (https://meet.jit.si/). We decided to try Jitsi because it had local popularity given reports that it was easy to log in once the app was downloaded on a phone, and it works with low bandwidth network coverage. CHVs were asked be in touch on the group WhatsApp if they were unable to install. The goal was for everyone to attempt connecting on Jitsi the following day, using a Jitsi meeting link.

One CHV was very proficient at Jitsi and helped the other CHVs with download, providing support via WhatsApp. Extracted WhatsApp chat shows some of the challenges the CHVs experienced, including wondering if there might be problem with the mobile phone itself or that the mobile phone did not have enough space/memory to download the App. However, by the end of the day, nine other CHVs were able to download Jitsi. The remaining CHVs were told to continue trying to download and update the others on their progress. The second “practice meeting,” this time using Jitsi, was set for the next day (October 8, 2020).

On our second practice Jitsi meeting, only one CHV had logged in. One challenge our team encountered was that when a group member could not use the provided link to join the meeting and shared this information on the group chat, other members would try to forward the link, creating challenges (and even sending different links). Members were asked not to forward links going forward, which helped.

**Figure 3.**
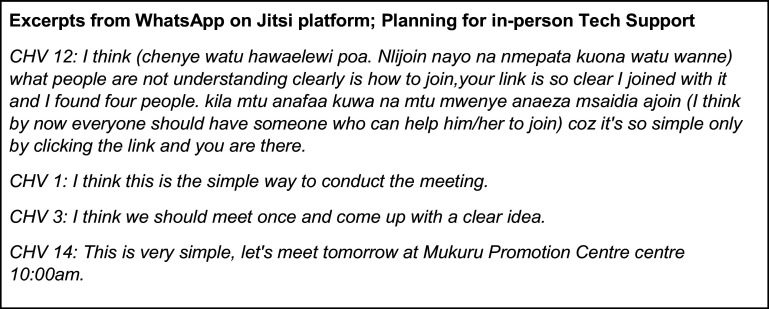
Excerpts from WhatsApp on Jitsi platform; Planning for in-person Tech Support.

**Step 4. Discuss and agree on the best platform and plan to support all trainees.** Trainees may have different experiences with platforms under consideration, and even with finding a platform that most can use, some may still experience challenges and need in-person support to download and join a virtual meeting. However, given that some will have success in Step 3, in most situations, these individuals can support colleagues/other trainees who need “hands on” or in-person support to download and join. In our project, as described below, this peer support was essential to success (See Figure 3).

In EQUIP, the group chat facilitated problem-solving among CHVs, and those who had challenges agreed that they would meet in a hall at the Mukuru Promotion Centre so that CHVs with challenges could be assisted by a proficient CHVs. All participants had to observe the government COVID-19 guidelines, wear masks, sanitize and maintain distance, with no more than five people in the same space. Four CHVs joined together in a room at the Promotion Centre. We continued to have challenges with old links being used; we instructed everyone via WhatsApp to delete all old links. On October 12, the study team sent a new link, and most CHVs were able to join. While there were still some challenges, there was a group agreement that Jitsi would be the best platform.

**Activity 5. Send guidelines for joining/video use.** To have a successful training, where all participants can hear and engage in the training, setting established guidelines in advance is important, particularly for those new to joining group virtual spaces, where being able to mute and unmute and reduce background noise can disrupt audio quality. Additionally, other preparation factors can facilitate a smooth virtual training, including ensuring charged devices (the mobile phone) and needed internet/data credit for the training duration, as well as guidance on when to use one’s own camera and not, given the bandwidth demands for camera use. For example, potentially the camera should be turned off except when a participant is asking questions or practicing a skill that needs to be observed.

**Figure 4.**
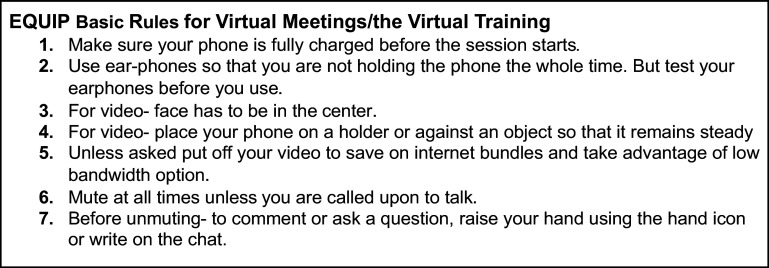
EQUIP Basic Rules for Virtual Mettings/Virtual Training.

In EQUIP, our research team sent the following guidelines to all training participants via our WhatsApp group chat. The guidelines were co-developed with the CHV participants. In the final training practice meeting, on our chosen platform (Jitsi), following these guidelines would be practiced (see Figure 4).

**Activity 6. Final practice meeting on chosen platform**. As a final step before the actual training date, it is beneficial to have trainees join the chosen platform, at an expected day and time, allowing for an opportunity to practice joining and the established guidelines. This way, the actual training can focus on content, mostly free from technology challenges

On October 16, 2020, 11/17 CHVs were able to join the meeting without help, using the provided link. Members were asked to add their names, allowing practice of adding names so that this could be done successfully in the actual training and facilitate better trainer/trainee connections. The next week allowed the participants to practice among themselves. The week also had a national holiday and some participants mentioned that they participate in community activities.

## EQUIP training in a mental health intervention

Using our six-step process allowed for successfully using Jitsi to train the CHVs, beginning on November 4, 2020. All CHVs joined and there were happy expressions of connection success, shared in the group WhatsApp chat (e.g., CHVs 8 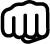 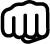; CHVs 2 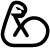).

Given CHVs need to fit training in around their other duties, we offered 4 h of training in the morning and 4 h of repeat training in the afternoon, for 5 days. The content for morning and afternoon sessions was the same, allowing CHVs to choose the time that was most convenient for them. This also allowed for smaller training groups and enhanced interaction between trainers and trainees. While there were normal disruptions that are common in virtual meetings (e.g., network issues in which connection was lost momentarily or power failure), CHVs who experienced disruptions during the morning sessions were able to make up in the afternoon. This was an additional benefit of our offering the same training content twice each day. We also offered one-on-one telephone support to cover for any missed sessions, which seemed to be an essential support for remote training when the internet is not consistently reliable.

## Discussion

Most efforts focusing on remote training rely on high-tech solutions (computers, high-speed internet) (Roland et al., 2020). There are fewer guidelines or training options for lower-tech contexts, where only mobile phones are available. Due to burgeoning smartphone use, remote training that can be delivered via smartphone offers great potential for broader training access. We were able to successfully train CHVs using smartphones; however, these efforts were not without challenges. Engaging CHVs in planning and support activities that occurred prior to the training itself was crucial to our success. Importantly, though we conducted the training online, offering an in-person option to support those who continued to experience challenges with logging onto the training platform was also important to ensure all CHVs were able to access the training.

The COVID-19 pandemic presented a situation where projects and organizations had to stop operating or adapt to a new mode of doing things, such as adapting virtual solutions. While WhatsApp had been well accepted and used extensively as an information and social/entertainment platform in Kenya, virtual meeting platforms like Zoom, Google Meet and Jitsi among others for meeting and training were less common. Research has documented the importance of training CHVs in new technologies (Ngabo et al., 2012; Feroz et al., 2020). A study by Bakibinga et al. (2020) looking at challenges and prospects for implementation of CHVs’ digital health solutions in Kenya, identified challenges with smartphones as an issue. Other work with lay counselors delivering a mental health intervention in Bungoma, Kenya has also outlined the challenges that lay counselors face when using mobile phones for other clinical purposes, like delivering or receiving supervision, including limited access to smartphones, challenges learning and using WhatsApp or mobile network connection disruptions (Triplett et al., 2023). In our study, the CHVs had smartphones; however, most had only used them to send and receive WhatsApp messages. They had never used the many other features included, and even downloading a new application proved to be challenging. However, with support and *built in time* (i.e., nearly 6 weeks) for technology attempts, collaborative decision-making, practice, having questions answered well before they needed to use the technology to attend training, our mobile phone training was successful.

Use of mobile phones for training can be a useful tool as we discovered, not only in times of pandemics but also in normal situations where distance, transport and traffic are a concern and cost that can be a prohibitive barrier. Considerations of each geographical area are important. For example, in rural areas, where distance and transport are concerns, CHVs may be less likely to all have smart phones and project support would need to include providing smartphones to those without them to enable use of this training approach (Triplett et al., 2023). We believe this approach is a valuable and low-cost way to provide some/all training even in a non-COVID context. We are, however, of the opinion that there may be a need for at least one in-person meeting like the one carried out at the Mukuru Promotion Centre (Activity 4), to ensure that all are comfortable with the virtual platform. Even with this one meeting, in our experience, this approach still greatly cuts down on the various costs associated with in-person training or virtual training that requires computer and high bandwidth access. We hope others can use our six-step process for their own trainings, and add to practical examples and supports in the literature for low-technology, virtual training solutions.

## Conclusion

Training CHVs to deliver a psychosocial intervention using smartphones is possible. The lesson from the team is that even in difficult times, available resources can be utilized for training and this ensures that program implementation does not stall. As a group, however, it would be important to establish the easiest and most affordable method when resources are constrained. The goal of this study was not to evaluate the effectiveness of telephone-based training. However, with the possibility using telephone as a median of training, this can be an important area of a future study.

## Data Availability

This was a qualitative section with no data.
