# Supplementary material for: Training community health volunteers to use mobile platform during the COVID-19 pandemic: The Kenya experience
Source: Glob Ment Health (Camb). 2024 Mar 22;11:e38. doi: 10.1017/gmh.2024.35 (PMC11016365; doi:10.1017/gmh.2024.35)
Supplement: Peer review history

# Editor report: Recommendation: Training community health volunteers to use mobile platform during the COVID-19 pandemic: The Kenya experience — R0/PR2

**DOI:** 10.1017/gmh.2024.35.pr2

**Affiliations:** University of Zimbabwe Faculty of Medicine, Zimbabwe

Thank you for submitting this important well written paper on utilising digital training to conduct training with community health volunteers. I have highlighted a few comments below:

1. In the paper, the abbreviation CHV is used for community health workers. Is it supposed to be community health volunteers (CHV) or community health workers (CHW)

2. Please confirm if Figure 1 is the same as the 4 yellow diagrams in the paper?

3. Please provide a brief description of the CHVs who participated in the training. Do they work just on mental health or they work in other health departments? How many years experience did they have as a CHV? etc

4.Please correct the following: Page 4 2nd paragraph line 17 “we wanted to start by determine participating CHVs”, add “if”. Page 4 3rd paragraph line 24, delete “we asked at the start of the sentence”. Page 5 3rd paragraph delete the repeated word “that” in line 27. Page 5 line 24, “Four CHVs joined together, in room...” can be corrected to “....joined together, in a room....”.

# Editor report: Decision: Training community health volunteers to use mobile platform during the COVID-19 pandemic: The Kenya experience — R0/PR3

**DOI:** 10.1017/gmh.2024.35.pr3

**Affiliations:** London School of Hygiene & Tropical Medicine, United Kingdom of Great Britain and Northern Ireland

No accompanying comment.

# Editor report: Recommendation: Training community health volunteers to use mobile platform during the COVID-19 pandemic: The Kenya experience — R1/PR5

**DOI:** 10.1017/gmh.2024.35.pr5

**Affiliations:** University of Zimbabwe Faculty of Medicine, Zimbabwe

No accompanying comment.

# Editor report: Decision: Training community health volunteers to use mobile platform during the COVID-19 pandemic: The Kenya experience — R1/PR6

**DOI:** 10.1017/gmh.2024.35.pr6

**Affiliations:** London School of Hygiene & Tropical Medicine, United Kingdom of Great Britain and Northern Ireland

No accompanying comment.
